# Do Children with Drug-Resistant Epilepsy Exhibit the Same Eating Habits and Feeding Difficulties as Their Healthy Peers? A Multifaceted Assessment of Eating Habits, Feeding Behaviors, and Nutritional Status in Pediatric Patients with Drug-Resistant Epilepsy on a Ketogenic Diet Compared with Healthy Children in Lower Silesia

**DOI:** 10.3390/nu18172788

**Published:** 2026-08-26

**Authors:** Katarzyna Daria Gołąbek, Agata Gruna-Ożarowska, Jakub Wronowicz, Bożena Regulska-Ilow

**Affiliations:** 1 Department of Dietetics and Bromatology, Pharmacy Faculty, Wroclaw Medical University, ul. Borowska 211, 50-556 Wrocław, Poland; bozena.regulska-ilow@umw.edu.pl; 2 Department of Pediatric Neurology, T. Marciniak Lower Silesian Specialist Hospital–Emergency Medicine Center, ul. Gen. August Emil Fieldorfa 2, 54-049 Wrocław, Poland; agata.ozarowska@wp.pl; 3 Statistical Analysis Centre, Wroclaw Medical University, ul. K. Marcinkowskiego 2-6, 50-368 Wrocław, Poland; jakub.wronowicz@umw.edu.pl

**Keywords:** ketogenic diet, drug-resistant epilepsy, eating behaviors

## Abstract

**Background/Objectives:** Age-related eating difficulties, combined with restrictive ketogenic diet therapies (KDTs), may contribute to a narrow range of consumed foods and reduced dietary variety in children’s diet. **Methods:** The study aim is to compare nutritional status, food neophobia (FN), food texture preferences, and eight eating behaviors—including food fussiness (FF)—as well as meal variety and repetitiveness, between children with drug-resistant epilepsy undergoing KDT (KD group; *n* = 15) and healthy children (*n* = 100), by the Child Food Texture Preference Questionnaire, the Children’s Eating Behavior Questionnaire and the Modified Children’s Food Neophobia Scale. **Results:** No significant differences were found in nutritional status (percentile: weight: 53 (31.50–78.00) vs. 51.50 (35.50–71.25), *p* > 0.9999; height: 43 (26.5–59.5) vs. 51 (31.75–70.25), *p* > 0.9999; BMI: 50 (21.5–92.5) vs. 51.5 (33–69.5), *p* > 0.9999) and in most eating behavior domains, although both groups showed a high prevalence of feeding difficulties. The KD group showed significantly greater meal repetition (3 (2–3) vs. 2 (1–2), *p* = 0.0468) and lower dietary variety (3 (2–3) vs. 4 (4–4), *p* = 0.0004), emotional undereating (4 (4–8.5) vs. 10 (9–13), *p* = 0.0059) and overeating (4 (4–7) vs. 8 (6–10), *p* = 0.0196) scores. Within the study population, eating behaviors clustered into distinct patterns predisposing toward either an “adaptive” or a “maladaptive” approach to food. **Conclusions:** There is a need for an individualized approach to addressing eating difficulties in children following KDT. It seems reasonable to introduce targeted screening assessments for FN and FF to facilitate early identification of patients at risk of unfavorable eating styles.

## 1. Introduction

The ketogenic diet (KD) has been used as a treatment for drug-resistant epilepsy since approximately 1921 [1]. Its aim is to induce an increase in serum ketone body concentrations owing to their antiseizure and anti-inflammatory properties and their potential neuroprotective effects [2]. Elevated concentrations of acetoacetate (AcAc), beta-hydroxybutyrate (BHB), and acetone (Ace) represent a physiological response to dietary changes, with values typically ranging from 0.5 to 5 mM/L [3]. Ketogenic diet therapies (KDTs) are a term encompassing the classic ketogenic diet (CKD) and its modified-ratio versions [4], modified ketogenic diet (MKD) [5], and several less restrictive variants: the modified Atkins diet (MAD) [6,7], medium-chain triglyceride diet (MCT) [8], and low-glycemic index treatment (LGIT) [9,10].

All these diets involve a significant reduction in carbohydrate intake, the maintenance of a standard amount of protein, and a substantial increase in fat intake relative to other nutrients. In the CKD, a specific ratio of fats and proteins to carbohydrates is calculated, e.g., 4:1, 3:1, 2:1, etc. The MAD proposed a strict approach to carbohydrate intake, limiting it to 20 g, while allowing greater flexibility in the consumption of fats and proteins [11]. Meanwhile, the MKD targets approximately 80% of energy from fat, with carbohydrate and protein amounts adjusted to improve dietary efficiency and meet the needs of patients [5].

The introduction of the KDT as a treatment for drug-resistant epilepsy has traditionally been recommended only after more than two antiepileptic drugs (AEDs) have proved ineffective. However, clinical specialists emphasize that the KDT should not be regarded as a last-resort treatment and should instead be introduced as soon as two AEDs have failed [11].

The effectiveness of the KDT is assessed as a reduction in seizure frequency of at least 50%. This outcome is observed in approximately 50–60% of patients with drug-resistant epilepsy of various etiologies. Approximately 10–15% of patients following the KDT become seizure-free [12]. Furthermore, children treated with KD showed improvements in cognitive function, learning ability, and sleep quality [13].

There is no maximum duration for KDT. The length of treatment depends on the patient’s condition and the benefits achieved. There are conditions, such as GLUT-1 deficiency syndrome (GLUT1-DS) or pyruvate dehydrogenase deficiency (PDHD), in which KDTs are maintained as a long-term treatment, extending into adulthood or continuing throughout life, typically with a gradually reduced level of dietary restriction. However, generally, it is recommended that diet continuation be thoroughly reassessed after 2 years, if it has proven effective, and that treatment be discontinued after 3 months if unsuccessful [11].

The restrictiveness of the KDT, including carbohydrate intake limitations, is difficult for patients to maintain and manage, and for parents to administer. Adherence to this regimen, particularly over the long term, is associated with increased perceived stress among caregivers [14]. However, it may also serve as a source of parental sense of agency through involvement in the child’s treatment. Both following the diet and the child’s illness affect the entire family [15].

During the introduction and maintenance of the diet, changes in eating habits and food preferences are observed among children, including the emergence of food refusal and food fussiness (FF) [16]. Additionally, KD products are often perceived by parents themselves as inconsistent with their children’s preferences [17].

It is worth emphasizing that childhood itself is associated with a higher prevalence of feeding difficulties in children, such as food neophobia (FN), a preference for only selected textures (soft or hard) [18], or picky eating [19,20]. Their etiology is multifaceted and related not only to the characteristics of the rejected foods themselves but also to behavioral and social problems [21]. It is also significantly influenced by parental food preferences, socioeconomic conditions [22,23], and sensory sensitivity [20].

Food neophobia and picky/fussy eating are two feeding strategies oriented toward food rejection and reduced food intake and dietary variety [24]. FN is characterized by a reluctance to try and consume new, unfamiliar foods, as well as a tendency to avoid them [25,26,27]. This strategy can be observed across all age groups. However, its peak prevalence is recorded at approximately 2–6 years of age [25].

Picky/fussy eating habits are characterized by the consumption of an inadequate amount and the quality of foods, both familiar and novel [28,29]. The amount of food consumed is often perceived as “poor” or “too small,” requiring a special approach by the family [30]. Fussy eating is associated with the emotional climate during mealtimes and increasing levels of stress and frustration among parents, who modify their feeding tactics and meal composition to “circumvent” the child’s feeding difficulties.

Although both strategies (FN, FF) are oriented toward avoidance, food neophobia appears to be more strongly associated with genetic factors, while picky/fussy eating appears to be more closely linked to socioeconomic ones [28,31].

In addition to meal- or product-related selectivity, children may also opt for specific food textures. It has been found that children with a tendency toward FN prefer softer foods that require less chewing [32]. Children give more favorable sensory ratings to, and are more willing to try, products whose texture and characteristics they perceive as “familiar” [33].

The impact of FN and FF on children’s height and body weight is unclear. Differences in the definitions used across studies prevent the identification of a precise effect on body weight consistently replicated in subsequent trials [33]. Some studies have observed a possible adverse effect of FN and FF on body weight or height compared with peers who do not exhibit selective eating behaviors [23]. However, it seems that a more significant aspect may be qualitative dietary deficiencies resulting from a narrow range of foods consumed [34,35]. In a Polish population of healthy children, a significant reduction in vegetable intake in the group of children with neophobic tendencies was observed [36].

Following a KDT may be associated with mineral and vitamin deficiencies due to the limited range of foods available for consumption. Particular attention is paid to bone health among children undergoing this treatment [11,37]. An inadequately balanced KDT may affect children’s growth. However, an increasing number of studies suggest that properly balanced therapies have no adverse effect on this parameter [38,39,40]. Nevertheless, some reports have noted growth impairment in children undergoing KDT compared with peers on a mixed diet [41,42]. The observed abnormalities may result from both possible micronutrient deficiencies and incorrectly implemented calorie restrictions, aimed at achieving higher concentrations of ketone bodies and thereby improving nervous system function [11].

Eating difficulties associated with childhood, combined with a restrictive KDT, may result in a limited range of foods being consumed. It appears that the long-term use of a diet accompanied by specific feeding-related limitations (FN, FF, food consistency preferences) may affect the quality of the diet and the nutritional status of patients.

Accordingly, the aim of the study was to compare nutritional status and dietary habits across multiple domains (dietary diversity, meal repetition, selective eating), food texture preferences (“soft” and “hard” texture likers), feeding-related difficulties, and eating behaviors (FN, FF, emotional overeating and undereating, food responsiveness, enjoyment of food, desire to drink, slowness in eating, and satiety responsiveness) between children with drug-resistant epilepsy following a KDT and their healthy peers, in order to identify the potential key differences between groups that may characterize their eating styles.

Furthermore, the study aimed to identify “clusters” of habits formed by correlated eating behaviors that may potentially influence dietary quality through children’s food choices.

Additionally, the study employed available validated instruments for assessing behavioral eating habits in healthy children to evaluate their potential applicability as screening tools for children with drug-resistant epilepsy.

## 2. Materials and Methods

### 2.1. Description of the Study

The study included two groups of children: a case group (ketogenic diet group–KD group)—children with drug-resistant epilepsy receiving ketogenic diet therapies—and a control group—healthy children following a mixed diet. One parent accompanied each child participant in every group, forming parent–child dyads. Anthropometric measurements were obtained for all pediatric participants, and BMI was subsequently calculated. All participants, together with a parent, completed a set of selected, validated questionnaires assessing behavioral eating patterns under the supervision of a qualified dietitian. The assessment encompassed the prevalence of FN, food texture preferences, eight distinct children’s eating behaviors (e.g., FF, slow eating), meal variety, meal repetition, and the occurrence of periods of selective eating. To evaluate differences in nutritional status, as well as the prevalence and severity of the selected eating habits and eating difficulties between the groups, the collected data were subjected to statistical comparison. Furthermore, correlations among the study variables and the clustering of eating habits were also assessed.

#### 2.1.1. Inclusion and Exclusion Criteria for the Group Following KDT

The entire population of pediatric patients with drug-resistant epilepsy (*n* = 22) undergoing KDT at the only public hospital in Lower Silesia offering this type of treatment—namely, the T. Marciniak Lower Silesian Specialist Hospital–Emergency Medicine Center in Wrocław, Poland—was invited to participate in the study.

The inclusion criteria for the KD group were as follows: consent from the child and their carers to participate in the study; a diagnosis of drug-resistant epilepsy and at least 3 months’ adherence to the ketogenic diet therapies; age > 2 years; and adherence to a fully or partially oral diet (to the extent that the child was able to express their dietary preferences).

The exclusion criteria were as follows: lack of consent from the child and their carers to participate in the study (*n* = 3; rejection rate = 13.6%); non-adherence to KDT recommendations; 100% enteral feeding via PEG (*n* = 2); age below 2 years (*n* = 2); treatment at a clinical center outside of Lower Silesia; and inability to perform anthropometric measurements.

The data were collected between October 2023 and February 2024.

#### 2.1.2. Inclusion and Exclusion Criteria for the Control Group

The study assumed that the control group should be representative of the KD group in terms of age (range 3–15 years) and place of residence (53.33% in areas with a population of over 100,000; 46.67% in areas with a population of under 100.000). To this end, a total group of 100 residents of Lower Silesia was formed. Fifty per cent of them lived in Wrocław (the capital of Lower Silesia; >100,000 inhabitants), whereas 50% resided in 16 various towns in Lower Silesia with <100,000 inhabitants. Each group (*n* = 50), divided according to place of residence, comprised three age subgroups, corresponding to the age distribution of the study group: younger children aged 3–6 attending kindergarten (32% of respondents), children in the early years of primary school aged 7–10 (32% of respondents), and adolescents aged 11–15 (36% of respondents). Each age subgroup included at least one child representing each year within the subgroup’s age range. For example, the 3–6-year-old group included children aged 3, 4, 5, and 6. Furthermore, an even distribution by sex was ensured in each age subgroup.

The inclusion criteria for the control group were as follows: consent from both the parent and the child to participate in the study; no chronic illnesses; no long-term medication use; no assessments from a psychological counseling center (e.g., dyslexia, dysgraphia, social interaction disorders); no diagnoses such as Asperger’s syndrome, autism, or other autism spectrum disorders or neurological disorders; the ability to undergo anthropometric measurements; Polish citizenship; and residence in Lower Silesia.

The exclusion criteria were as follows: refusal to consent to the study; chronic illnesses, including neurological disorders; diagnoses from a psychological counseling center; adherence to specialized diets related to a medical condition or exclusionary diets (vegetarian, vegan, ketogenic); long-term use of medication related to a medical condition; and inability to perform anthropometric measurements.

Healthy controls were selected based on the inclusion criteria and matched to the case group according to place of residence, age, and sex.

### 2.2. Anthropometric Measurements

Body height and weight were measured in all pediatric participants. Height was measured using a free-standing, portable Tanita HR-001 stadiometer (Tanita, Tokyo, Japan), and weight was measured using a Tanita BC-545 scale (Tanita, Tokyo, Japan). Measurements were performed by the researchers for 85% of the participants. For the remaining 15% (healthy children only), data were provided by parents based on measurements taken by a sports medicine physician or recorded by the family physician no more than 2 months prior to the study visit.

Based on the collected data, BMI was calculated and compared against the Polish percentile charts derived from the OLA and OLAF studies—two nationwide research projects (OLAF, PL0080, and OLA, NR13 0002 06) that established growth and blood pressure references for the Polish pediatric population. In accordance with current recommendations, the following cut-off points were adopted: BMI < 5th percentile was classified as underweight, 5th–85th percentile as normal weight, >85th percentile as overweight, and >95th percentile as obesity [43].

### 2.3. Questionnaires Assessing Feeding Habits and Difficulties

To assess behavioral eating patterns, parents and children from the study groups, together with a qualified dietitian, completed the following questionnaires: the Modified Children’s Food Neophobia Scale (Modified CFNS) [44,45,46] (parent reported), the Children’s Eating Behaviour Questionnaire (CEBQ) [47,48] (parent reported), and the Child Food Texture Preference Questionnaire (CFTPQ) [32,49] (child reported), all translated into Polish.

#### 2.3.1. Modified Children’s Food Neophobia Scale

The Modified CFNS was used to assess the prevalence and severity of food neophobia. This parent-completed questionnaire consisted of 6 items, each scored on a 1–4 scale according to the extent of parental agreement with the statements, with two items reverse-scored. A total score of >12 points was considered indicative of a higher degree of food neophobia, while scores ≤ 12 points indicated lower food neophobia [45,46]. Following other authors, values above the M + 1SD, calculated for the study population, were used as the cut-off for diagnosing neophobia [34,50].

#### 2.3.2. Children’s Eating Behavior Questionnaire

The CEBQ [51] is a parent-report questionnaire consisting of 35 items assessing children’s eating style. The questionnaire evaluates eight scales: food responsiveness (4 items), enjoyment of food (4 items), emotional overeating (4 items), desire to drink (3 items), satiety responsiveness (5 items), slowness in eating (4 items), emotional undereating (4 items), and fussiness (7 items). Each item is rated on a 5-point frequency scale, from 1 (never) to 5 (always), with 5 items reverse-scored. As with the FN assessment, scores above 50% of the maximum possible score for a given subscale were considered indicative of an elevated representation of that eating style, whereas scores above the M + 1SD were considered indicative of a high representation of that eating style. The questionnaire has been validated for use in the Polish population [52].

#### 2.3.3. The Child Food Texture Preference Questionnaire

The CFTPQ was completed by children. The tool involved selecting the preferred texture of a given food product from a pair of options. The results were converted into a CFTPQ index: CFTPQ index $=\left[\left(\frac{Sum\;of\;the\;scores\;of\;the\;valid\;pairs}{Total\;number\;of\;valid\;pairs}\right)-1\right]*100$, ranging from 0 to 100 points. Following the authors of the questionnaire, scores below the 25th percentile (Q1) were classified as indicating “soft likers”, while scores above the 75th percentile (Q3) were classified as indicating “hard likers”. The questionnaire is suitable for use in children from European countries [32].

#### 2.3.4. Additional Questions

To complement the information on eating style, parents of children in both study groups were asked additional questions regarding meal diversity, meal repetitiveness, and the occurrence of periods of selective eating.

1. Has your child experienced periods of selective eating?
  (a) Yes (1 pt)
  (b) No (0 pt)
2. Which food groups/meals were most frequently rejected by your child during these periods? (e.g., vegetables, fruits, dairy products, meat, fish, eggs; PLEASE LIST 3–4 MOST OFTEN OMITTED)?
3. Are there days when your child eats the same dish for all meals during the day?
  (a) Yes (1 pt)
  (b) No (0 pt)
4. How often is the same type of meal repeated during one week?
  (a) 2 times (1 pt)
  (b) 3–5 times (2 pts)
  (c) 5–8 times (3 pts)
  (d) more than 8 times (4 pts)
5. Are there weeks during which your child consumes?
  (a) A maximum of 3 different meals during the week (1 pt)
  (b) A maximum of 5 different meals during the week (2 pts)
  (c) A maximum of 8 different meals during the week (3 pts)
  (d) The child usually eats a varied diet (4 pts)

### 2.4. Statistical Analysis

The results obtained from the study groups were compared to assess significant differences between them. Statistical analyses were performed using R software (version 4.6.1). Normally distributed variables were presented as mean ± standard deviation, while non-normally distributed variables as median (interquartile range).

The normality of distribution was tested using the Shapiro–Wilk test when the number of participants in a group was ≥10. The homogeneity of variances was assessed by Levene’s test. The Mann–Whitney U test was performed for continuous variables with a non-normal distribution, and the Chi-square test for categorical variables. For most continuous variables, the Mann–Whitney U test was used. For sex and the prevalence of food selectivity, Pearson’s Chi-squared test was employed, and Fisher’s exact test was used in cases where at least 20% of the expected cell counts were less than 5. To assess differences in BMI percentiles and nutritional status between groups by sex, two-way ANOVA was applied after confirming the homogeneity of variances with Levene’s test, along with a non-parametric Aligned Rank Transform ANOVA using the ARTool package. To assess the differences in the prevalence of neophobia levels, Fisher’s exact test was performed, and to assess differences in the prevalence of “soft”- and “hard”-texture likers, Pearson’s Chi-squared test.

A Bonferroni correction for multiple testing was applied whenever more than one test was considered within one family of hypotheses. The statistical significance was defined as an adjusted *p*-value < 0.05.

Spearman’s rank correlation analysis was also performed to assess associations between age, nutritional status, and eating behavior variables in the study population.

Principal Component Analysis (PCA) without rotation or kernel transformation was conducted to identify potential patterns among the continuous variables. The input data has been centered and scaled to ensure that variables measured on different scales contributed comparably to the analysis.

## 3. Results

### 3.1. Baseline Characteristics and Nutritional Status of the Study Populations

The KD group included 15 participants who fully completed the study. The control group included 100 participants. In the KD group, 40% of participants (*n* = 6) were younger than 7 years, 33.33% (*n* = 5) were aged 7–10 years, and 26.67% (*n* = 4) were aged ≥10 years. Similarly, in the control group, 32% (*n* = 32), 32% (*n* = 32), and 36% (*n* = 36) of participants were in the respective age categories. In the KD group, 73.33% of children followed CKD, 20% MAD, and 6.67% LGIT. In the control group, 100% of the participants followed a mixed diet with no dietary restrictions.

Table 1 presents a comparison of demographic and nutritional characteristics, including sex distribution, between the study groups.

No significant differences were observed between children in the KD and control groups for age, sex distribution, place of residence, or height and weight percentile values. Moreover, there were no statistically significant differences in the percentage of participants with underweight, normal weight, overweight, or obesity (*p* = 0.0396; adjusted *p* = 0.9499).

Also, there were no significant differences in BMI percentile (two-way ANOVA; F = 0.769, *p* = 0.3821), and nutritional status (two-sided Fisher’s exact test, *p* = 0.0637) between sex groups in either the control or KD groups.

### 3.2. Eating Behaviors, Habits, and Food Selectivity

#### 3.2.1. Food Neophobia

Based on Modified CFNS results presented in Table 2, a higher degree of neophobia (>12 pts) was found in approximately 60% of participants in the KD group and 73% of the control group. There were no statistically significant differences between the groups (*p* = 0.3605). The medians obtained between the groups were also not statistically different: 14 (9–17) vs. 15 (12–18) (min: 6 vs. 7; max: 23 vs. 24) (*p* = 0.2500; adjusted *p* > 0.9999). Neophobia (M + 1SD = 18 pts) was identified in 13.33% of children undergoing KDT and 19% of healthy children.

There were no significant differences between the groups in the association between neophobia level and body weight (Aligned Rank Transform ANOVA using the ARTool package; F(1.111) = 0.99, *p* = 0.3210), height (F(1.111) = 0.74, *p* = 0.3930), or BMI (F(1.111) = 0.48, *p* = 0.4880).

#### 3.2.2. Food Texture Preference

Based on data obtained from the studied children, food texture preferences were compared between the groups, with results presented in Table 2, The CFTPQ index (min. 0 pts; max. 100 pts) showed no statistically significant difference between the groups in median values: 47.06 (29.41–61.76) vs. 52.94 (47.06–64.71) (min: 17.65 vs. 11.76; max: 76.47 vs. 100.00) (*p* = 0.1491; adjusted *p* > 0.9999). Based on Q1 and Q3, the percentage of “soft-texture likers” (<25th percentile) was set at 40% of participants in the KD group and approximately 24% in the control group. Similarly, “hard-texture likers” (>75th percentile) accounted for 33.33% and 40% of participants, respectively. There were no statistically significant differences between the groups (*p* = 0.4159).

#### 3.2.3. Children’s Eating Behaviors

Table 3 presents the assessment of eight children’s eating behaviors by CEBQ and the statistical differences between the KD and control groups.

There were no statistically significant differences between children in the KD and control groups for eating behaviors associated with food fussiness, satiety responsiveness, food responsiveness, desire to drink, and enjoyment of food. Significant differences were observed for eating behaviors related to emotional regulation, i.e., emotional overeating: 4 (4–7) vs. 8 (6–10) (*p* = 0.0008; adjusted *p* = 0.0196), and emotional undereating: 4 (4–8.5) vs. 10 (9–13) (*p* = 0.0002; adjusted *p* = 0.0059). The percentages of participants exhibiting low, elevated, and high levels of eating behaviors, as assessed by the CEBQ, are presented in Table 4 for both the control and KD groups.

#### 3.2.4. Selected Eating, Dietary Diversity, and Meal Repetition

To further explore eating behaviors and habits among the participants, the authors administered additional questions assessing dietary diversity and meal repetition across the groups.

##### Selective Eating

There were no statistically significant differences between the groups in the percentage of participants who had experienced at least one significant episode of selective eating during their lifetime: 64.29% (*n* = 9/14) vs. 39% (*n* = 39/100) (*p* = 0.038; adjusted *p* = 0.9131).

Parents of participants from the KD group who had experienced selective eating reported that, during these episodes, 71.43% (*n* = 5) of children excluded all vegetables from their diet. It was noted by 57.14% (*n* = 4) of parents that their child chose only 2–3 products and consumed them repeatedly, and 42.86% (*n* = 3) of children excluded high-fat products such as mayonnaise and fatty sauces.

Furthermore, in the control group, among the 51.28% (*n* = 20) of participants who experienced selective eating episodes, meat and meat products were excluded, followed by fish (28.20%) and eggs (15.38%). Similar to the children in the KD group, a high percentage of individuals from the control group excluded vegetables from their diet (76.92%, *n* = 30). Only 2.56% were characterized by choosing 2–3 products and consuming them repeatedly.

##### Meals Repetition

In both groups, parents reported some children who episodically consumed the same dish at every meal during a day. A higher percentage of children in the KD group exhibited this behavior compared with participants in the control group (23.08% vs. 5%). However, after correction for multiple comparisons, this difference was not statistically significant (*p* = 0.0200, adjusted *p* = 0.4741).

Nonetheless, there was a significant difference between groups in meal repetition frequency. Participants from the KD group obtained significantly higher scores (3 (2–3) pts, corresponding to 5–8 repetitions of the same meal per week) compared with the control group (2 (1–2) pts, corresponding to 3–5 reps/w) (*p* = 0.0019; adjusted *p* = 0.0468).

##### Meals Diversity

Similarly, based on parental reports, children following KDT presented significantly lower meal diversity compared with healthy ones. Participants in the KD group obtained 3 (2–3) pts, corresponding to 5–8 different meals consumed per week, while children in the control group obtained 4 (4–4) pts, denoting a greater weekly dietary diversity (i.e., more than 8 different meals) (*p* < 0.0001; adjusted *p* = 0.0004).

### 3.3. Correlations Between Eating Behaviors and Habits and Anthropometric Measurements

**Figure 1:**
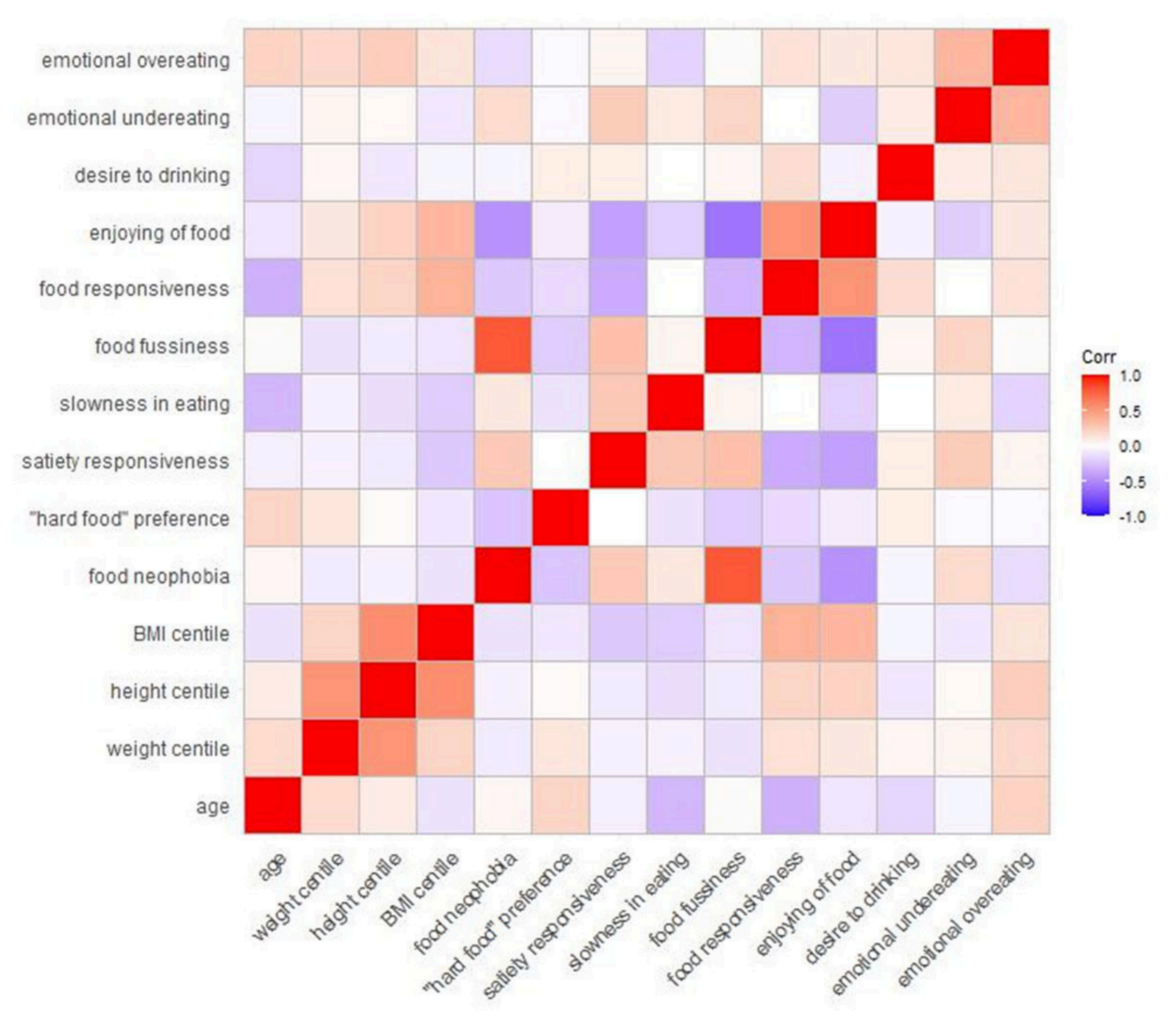
Spearman correlation analysis between age, nutritional status, and eating behaviors variables in the study population. Color intensity corresponds to the magnitude of the correlation (Spearman’s ρ); red indicates positive correlations and blue indicates negative correlations. Correlation coefficient.

**Figure 2:**
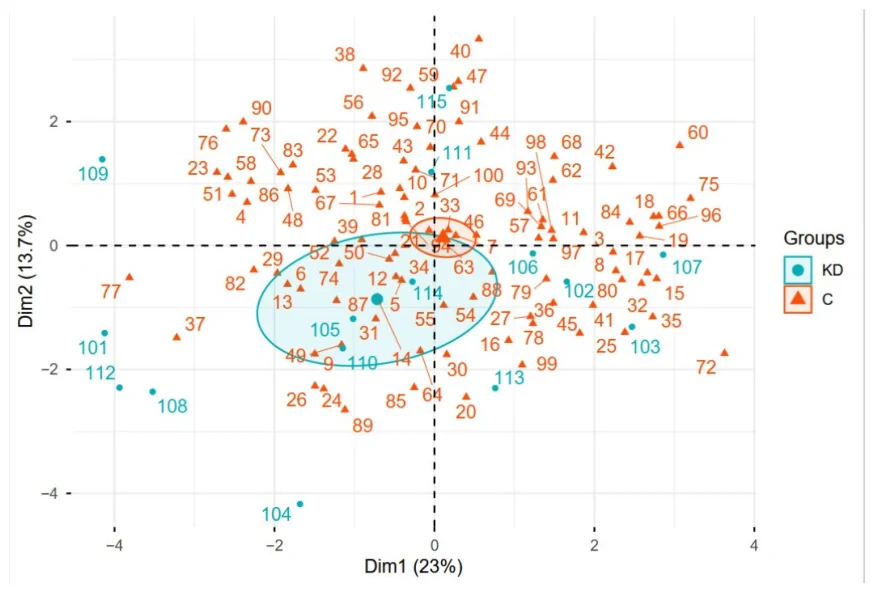
Principal Component Analysis (PCA) plot showing individual data points projected onto the first two dimensions (Dim1: 23% variance explained; Dim2: 13.7% variance explained). Colors indicate the groups: control (red) and KD (blue).

Figure 1 illustrates the correlation matrix among the studied variables. Considering the high degree of similarity observed between the KD and control groups, subgroup-specific analyses were deemed unnecessary. Therefore, correlation analyses were performed on the pooled study sample as a whole. Furthermore, this assumption was tested by adding a supplementary qualitative variable, group, as shown in Figure 2. The resulting plot showed no clear separation between the groups, indicating no significant differences in the analyzed variable profile.

The study further evaluated between-group differences in the prevalence of two distinct behavioral phenotypes: children with “complicated” eating patterns—defined by a higher level of FN (>12 pts), a preference for soft-textured foods (<25th percentile), and a high degree of FF (>22 pts)—and children with “non-complicated” eating patterns—defined by a lower level of FN (≤12 pts), an FF score within the normal range (≤22 pts), and a preference for hard-textured foods (>75th percentile). No statistically significant difference was observed in the prevalence of either phenotype (*p* = 0.3451).

### 3.4. Variables PCA

Principal Component Analysis (PCA) was applied to examine the correlation structure among the studied variables in the pooled study population (the KD and control groups combined).

**Figure 3:**
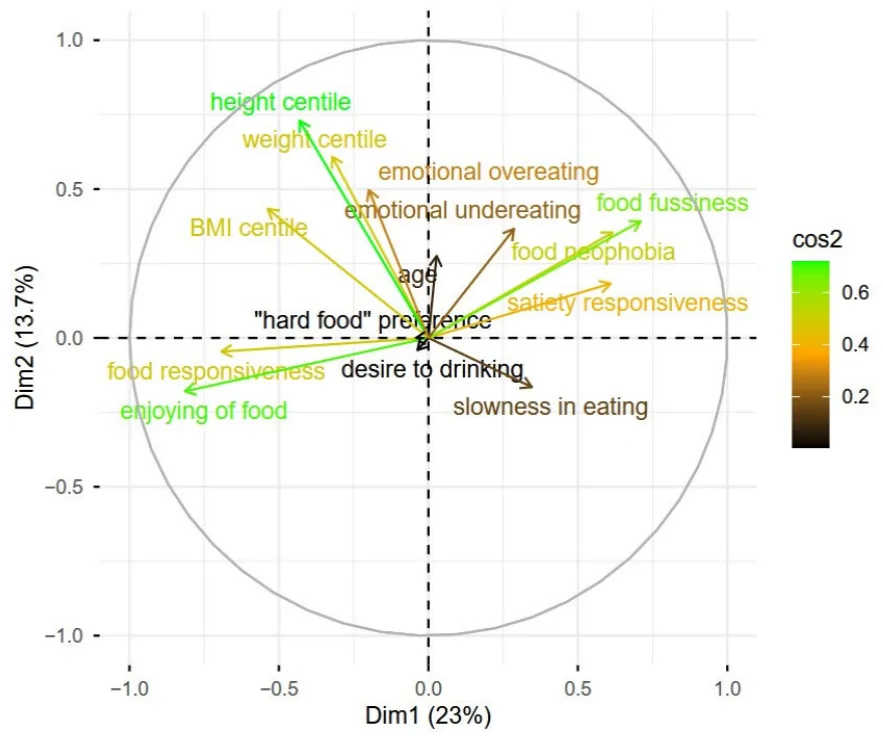
Principal component analysis (PCA) correlation circle of variables on the first two principal components (Dim1, Dim2). The color indicates the quality of the variable’s representation on the first two principal components.

**Figure 4:**
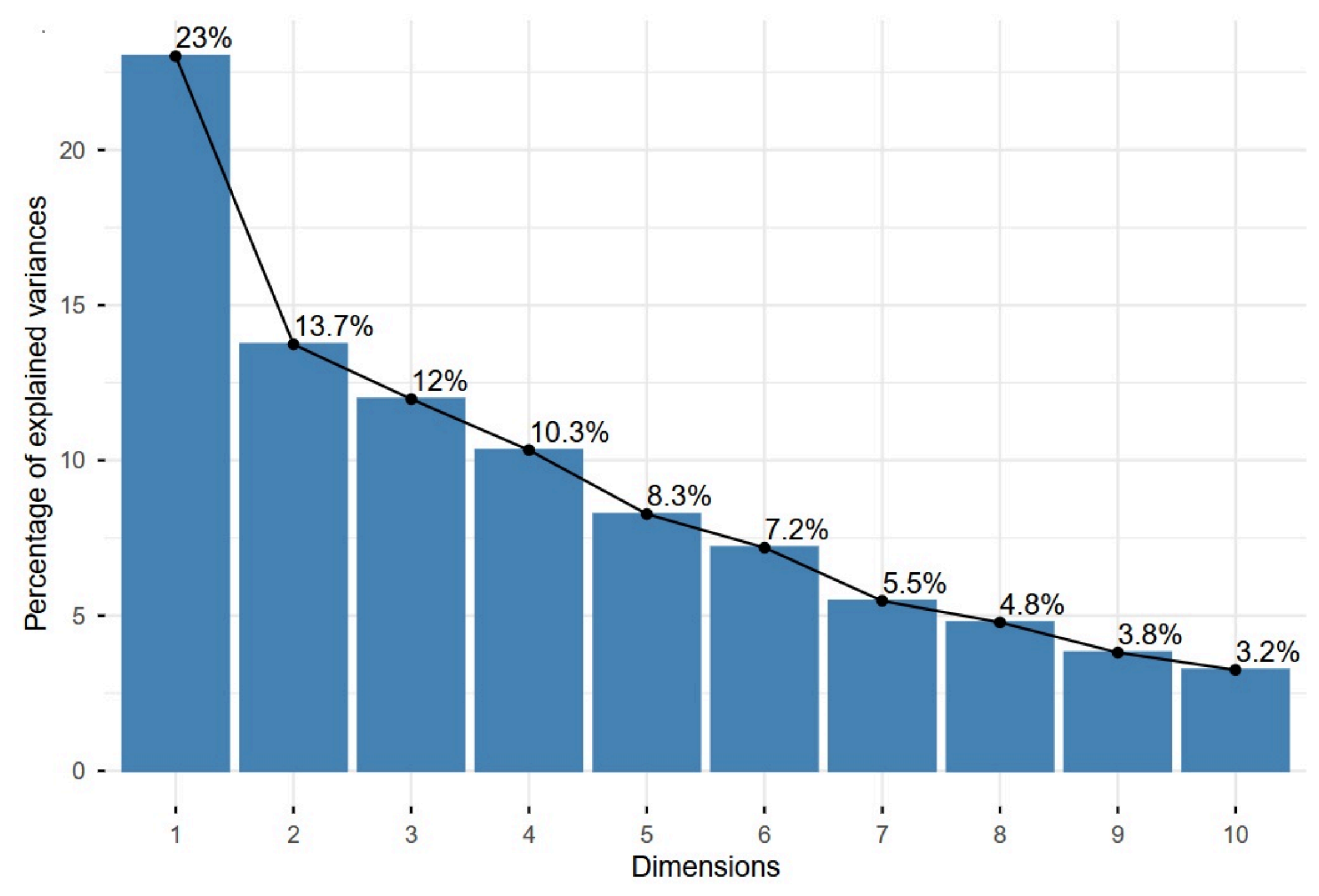
Scree plot of explained variance for each principal component.

Figure 3 presents the correlation circle of variables obtained from the Principal Component Analysis (PCA). Dimensions 1 (Dim1) and 2 (Dim2) explained 23% and 13.7% of the total variance, respectively, accounting for a combined 36.7% of the total variance, as shown in Figure 4.

**Figure 5:**
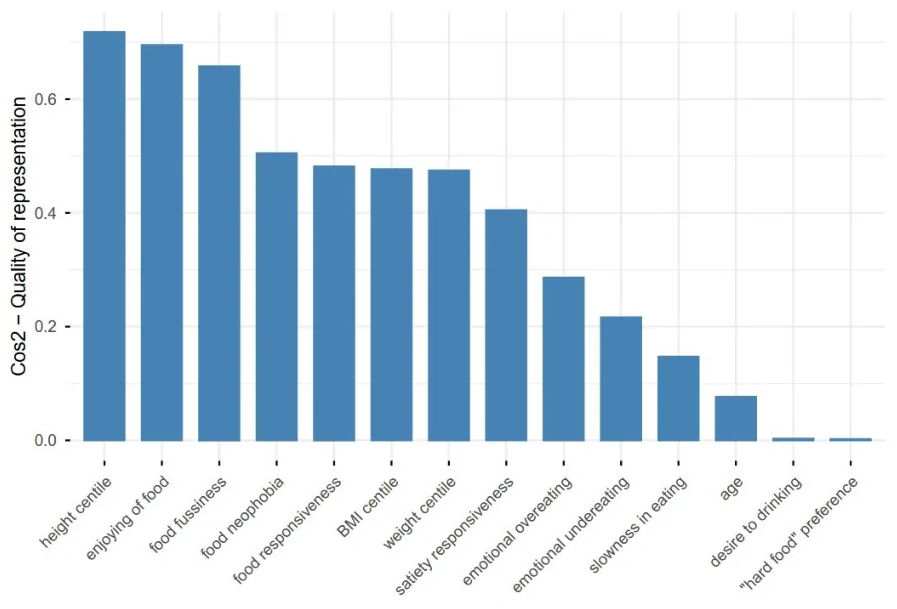
Cos^2^ of variables for the first two principal components.

Figure 5 presents the cos^2^ values of variables with respect to the first two principal components (Dim1 and Dim2), where higher cos^2^ values indicate a better representation of the variables within this two-dimensional space.

Three groups of interrelated and correlated variables were assessed:

(1) a group of habits associated with maladaptive eating behaviors—the “difficult group”—which consisted of food fussiness, degree of food neophobia, emotional undereating, and satiety responsiveness;
(2) a group of habits associated with adaptive eating behaviors—the “easy group”—comprising enjoyment of food, food responsiveness, and preference for hard-textured foods;
(3) an anthropometric correlation group consisting of emotional overeating, BMI, body weight, and height percentiles.

Desire to drink, age, and slowness in eating were not correlated with the other variables.

It was observed that the first component (Dim1) primarily distinguished the variables related to adaptive behaviors, as evidenced by the opposing positions of variables such as FF, FN, and satiety responsiveness relative to enjoyment of food, food responsiveness, and preference for hard food consistency. The “food fussiness” variable was characterized by a high positive loading, suggesting a strong inverse relationship with adaptive behaviors. Furthermore, the group of anthropometric variables was negatively associated with the group of maladaptive eating styles.

By contrast, enjoyment of food and food responsiveness showed negative loadings on Dim1, further emphasizing the inverse correlation between these two groups of variables. The second component (Dim2) primarily reflects anthropometric variables, as indicated by the high loadings of variables such as body weight, height, and emotional undereating. A strong positive correlation was found between FF and degree of FN, as well as a strong positive correlation between enjoyment of food and food responsiveness.

## 4. Discussion

The present study found no significant differences in nutritional status between children with drug-resistant epilepsy treated with the KDT and their healthy peers. BMI percentile values did not differ significantly. Similar to Han et al. [53] and Chang et al. [54], these findings suggest that a well-managed KDT does not necessarily lead to stunted growth or significant changes in children’s physical development. Mendonça et al. [39] observed that the KD may even serve as a tool to correct underweight in children. A meta-analysis of 11 studies [40] also found no significant changes in body weight during KDT.

In the present study, no statistically significant differences in height were observed between groups, either among boys or girls. Lu et al. [55] reported a possible attenuation of the growth curve in children undergoing KDT. Collectively, these findings suggest that longitudinal monitoring of growth linearity—both during and after ketogenic diet treatment—may represent the most clinically relevant approach to assessing nutritional status in this pediatric population.

Although the groups did not differ significantly in nutritional status, a higher percentage of children with overweight or obesity was noted in the KD group. A similarly high percentage of children with excessive weight on the KDT was reported by de Mendonça et al. [39] (33.33% in the present study vs. 28.6–41.2%). Adherence to a KDT requires careful monitoring and regular, systematic assessment of nutritional status. Nevertheless, a growing body of evidence suggests that the KDT may represent a safe dietary approach for children with respect to physical development.

Although children with drug-resistant epilepsy adhere to an atypical dietary regimen and live with a condition that distinguishes them from their peers, they experience dietary problems and difficulties comparable to those of healthy children. In the present study, no statistically significant differences were observed between the percentage of children exhibiting a higher (60% vs. 73%) or lower (40% vs. 20%) degree of FN in the KD and the control groups. A systematic review of 41 studies employing various assessment tools reported high FN levels, ranging from 40% to 60% among children aged 0–18 years [33]. These findings indicate that the results obtained in the present study are consistent with previously published data. Similarly, among Polish children aged 2–7 years, a heightened level of neophobia was identified in 40% of the sample, with no sex-related differences observed, as in the current study [56]. Comparable results were reported in a younger Irish cohort (1.5–3.4 years), where 47.80% exhibited elevated food neophobia scores [44]. By contrast, lower rates were observed in Chinese (1–3 years) and Italian (7–12 years) populations, at 23.7% and 25.1%, respectively. However, these findings were derived using a different tool, the Child Food Neophobia Scale, which may account for the noted discrepancy [57,58].

Furthermore, in another Polish population of children aged 3–7 years, also assessed using the Modified Child’s Food Neophobia Scale, the prevalence of FN was estimated at 10.8%, compared with 13.3% in the KD group and 19% in the control group [50]. Similarly, figures were reported in a review of 41 studies, with FN ranging from 14% to 16% [33].

A higher degree of FN represents a common dietary challenge during childhood, with a clear trend toward a decrease in prevalence with age [59]. Its elevated levels are associated with poorer dietary quality [34,60] and reduced vegetable intake [36]. Krupa-Kotara et al. [61] reported a positive correlation between a high degree of FN and lower BMI values. However, this association has not been consistently replicated across studies [62]. No such relationship was identified in either the KD or the control group in our investigation.

Therefore, it is important to closely monitor and assess the dietary intake and developmental status of children on a KD who exhibit neophobic tendencies. This is because the therapeutic diet itself inherently restricts the range of food choices available, and when combined with neophobia, may further compromise an already limited dietary variety.

Texture constitutes an important nutritional aspect of food, as well as a challenge with respect to chewing and food processing. Although 40% of children in the KD group exhibited a preference for soft textures, this percentage did not differ significantly from that observed in the control group (24%). Similarly, no differences were found in the percentage of “hard likers” between the groups (33.33% vs. 40%). Hard textures, such as those found in nuts, vegetables, and fresh fiber-rich foods, may pose a dietary challenge for an average of 30% of all children included in the study.

Similar values to those obtained in the present study were observed in a group of 70 healthy children from Denmark, aged 6–13 years, also assessed by the CFTPQ, of whom 31.4% were classified as “soft likers” and 35.7% as “hard likers” [49]. Comparable findings emerged from a study involving children from six European countries, evaluated with the same tool. Significantly higher percentages of “hard likers” were reported among Finnish and Swedish children (34.3% and 37.3%, respectively), consistent with the present results, whereas southern European populations showed lower percentages (18.3–23.3%). The prevalence of “soft likers” was reported at 10% of participants in Finland, 26.8% in Italy, 29% in Spain, and 36% in the UK [32].

As in the study groups, no differences were observed in European pediatric populations regarding sex, age, weight, or height based on texture preferences. Consistent with the present study, a modest negative correlation was found between a preference for hard textures and FN [32,49]. Furthermore, Danish children who preferred soft textures were found to exhibit significantly higher sensitivity to all sensory modalities of food, including touch, taste, sound, and smell [49].

The higher proportion of children in the KD group exhibiting a preference for soft textures, compared with healthy children, may potentially be attributable to early experiences such as tube feeding [63], reduced muscle strength required for mastication, and differences in nervous system function, including possible alterations in sensory processing [64]. However, it should be emphasized that the obtained values did not differ significantly from those of the control group.

Based on the findings, it is not possible to identify a single predominant texture preference among children on a therapeutic diet or healthy children. Our observations indicate the need to individualize dietary planning for both children on a mixed diet and those receiving KDT, ensuring adequate nutritional quality by incorporating preferred foods and progressively introducing novel textures to broaden the range of accepted products.

The influence of the underlying condition, early medical experiences, and the type and method of dietary weaning on texture preferences among children receiving KDT remains an unexplored area of research. Assessing this relationship seems particularly important, given that some children undergo this stage before the KDT, while others do so afterward [65].

In the present study, eight eating styles were assessed, four of which were classified as “food-approach subscales”—food responsiveness, emotional overeating, enjoyment of food, and desire to drink—and four as “food-avoidant subscales”—food fussiness, emotional undereating, satiety responsiveness, and slowness in eating [52]. Except for emotional overeating and undereating, none of the assessed eating behaviors differed significantly between the groups.

The control group’s food fussiness score (17.5) was comparable to values reported for children with normal weight (16) and overweight (15) aged 3–10 years in another Polish population. Children following KDT were characterized by a lower median value (13). However, this result did not significantly differ from that of the control group. The lower value observed in participants from the KD group may nevertheless stem from the restrictions imposed by the diet—particularly its classic variant—and the resulting inability to vary most meals [66].

It is nevertheless worth highlighting that, despite the dietary restrictions imposed by the KDT, 26.67% of participants from the case group exhibited elevated levels and 13.33% exhibited a high level of FF. Notwithstanding these dietary restrictions, the children in the present study demonstrated results that were consistent both internally and with those reported for another group of Polish children with respect to enjoyment of food [66].

It seems important to evaluate the occurrence of FF in the pediatric populations. For instance, a study by Masento et al. [67], which assessed pre-school children from Poland, the UK, and Italy, found that food fussiness—measured using the same scale as in the present study—was a predictor of reduced vegetable intake. The results of our investigation indicate that children with drug-resistant epilepsy do not differ from their healthy peers in terms of food fussiness levels. It is worth noting, however, that healthy children may have greater opportunities to express this behavior owing to greater dietary freedom, but both groups achieved similar results.

In the study by Malczyk et al. [66], significantly lower “slowness in eating” scores were observed in children with obesity compared to children with normal body weight (10 vs. 12). Similarly, although the difference did not reach statistical significance, scores of 10 and 11 were recorded in the control and KD group, respectively. It is possible that the higher scores observed in the KD group, relative to healthy children, may be linked to two factors: first, the sense of satiety achieved through the consumption of high-fat meals and the correspondingly slower pace of eating; and second, the potential mismatch between dietary preferences and the foods available within the KDT framework, which may prolong meal duration as a consequence of diminished palatability.

However, the results for satiety responsiveness showed no significant differences between children undergoing KDT and those in the control group, although participants in the case group exhibited lower values for this indicator. This may be attributable to smaller meal portions, compared with those of healthy children, and the inability to leave part of a meal uneaten, even when feeling full. The questionnaire assessed satiety responsiveness based on information regarding leaving portions on the plate and finishing a meal early when feeling full. Children on strict KDT may be less able to adjust their intake in response to satiety cues. It is worth noting that, in the study by Malczyk et al. [66], significantly lower satiety responsiveness was observed in individuals with obesity compared with those with normal body weight.

In the present study, we observed significantly lower food intake regulation in response to emotional experiences in the KD group. The KDT, especially CKD, is strict with respect to its composition, number of meals, specific mealtimes, and meal frequency. Owing to these dietary restrictions, children on KDT are less able to regulate their emotional expression through increased or decreased food intake. Healthy children—owing to greater dietary freedom—scored significantly higher than children with epilepsy in emotional under-eating (10 vs. 4) and over-eating (8 vs. 4). No such differences were observed in another Polish population of children with normal and excessive body weight, where scores for these eating behaviors were comparable (10 vs. 10 and 9 vs. 7 pts) [66].

A further question is whether, owing to their inability to express emotions through food, children undergoing KDT exhibit alternative behaviors that regulate and reflect their emotional state. Behavioral changes have been noted while following a KDT [68,69]. However, it remains unassessed whether these changes result from alterations in neuronal metabolism and neurotransmitter release, from a particular stage of child development, or indeed from changes in emotional expression itself or its behavioral regulation.

On the other hand, dietary restrictions that preclude the use of food as a reward or as a means of emotional regulation may reduce the occurrence of emotional eating in children by compelling parents to adopt alternative strategies and attitudes toward food consumption [70,71].

The present study showed that both study groups achieved low scores on the “desire to drink” scale (approx. <50% of points), indicating a need to monitor water intake and hydration status among children, regardless of underlying medical condition. In the majority of participants, limited interest in drinking and a reluctance to express a desire for hydration reflect a tendency not to meet hydration requirements—a pattern observed both in Polish children and in children from other European countries [66,72].

Both study groups experienced episodes of selective or picky eating. Although a higher percentage of participants in the KD group reported such episodes (69.23% vs. 39%), this difference did not reach statistical significance. These findings suggest that selective eating is a common experience during childhood [73].

The interrelationships between the assessed indicators are important in predicting children’s dietary choices. The PCA identified two interrelated behavioral clusters that may potentially influence dietary adherence. The adaptive eating behaviors group (“easy group”) comprises enjoyment of food and food responsiveness (strong correlation), together with texture preference (weak correlation). These traits may be predictive of children who are likely to accept dietary and meal changes more readily, show greater interest in food, and prefer firmer textures (e.g., vegetables and fruit) [74]. The higher the expression of food enjoyment and food responsiveness, the more plausibly this pattern is to be observed. These are potentially children who are interested in food, eager to eat, and willing to try new foods.

The second—the maladaptive eating behaviors group (“difficult group”)—is, by contrast, characterized by food fussiness and the degree of food neophobia (strong correlation), satiety responsiveness (moderately strong correlation), and emotional under-eating (weak correlation). High expression of these eating styles suggests that these children will find it more difficult to adapt to a diet and to any subsequent dietary changes. Furthermore, they may restrict the range of meals [75,76] included in their diet, particularly those containing foods they dislike or that trigger a phobic response [61,74]. Children who reach satiety more quickly may nevertheless have to finish their ketogenic meals despite feeling full, which may make dietary adherence more difficult for both children and their parents.

Furthermore, children following KDT consumed a significantly less varied diet over the course of a week, compared with healthy children, and repeated the same meal significantly more often. In light of our findings, it seems important to introduce screening for FF (the strongest variable within the “difficult” cluster)—for example, using the CEBQ Food Fussiness subscale—in order to identify patients likely to experience greater-than-usual difficulty in maintaining a nutritionally adequate and varied KDT. This is particularly relevant, given that 13.33% of patients in the KD group exhibited strong food fussiness and 26.67% an elevated level of food fussiness, together with 13.33% classified as neophobic and 46.67% exhibiting a higher degree of neophobia. Such patients with “maladaptive eating patterns” may constitute a substantial percentage of those under clinical care. This is a critical consideration, as a diet that is already inherently restricted may predispose to further monotony in these children, thereby contributing to inadequate intake of essential nutrients and to adverse health consequences.

Furthermore, individual dietary exclusions may limit the consumption of hard-textured foods, which stimulate the oral musculature [77] and speech development [78,79]. These findings also indicate the need to monitor both meal repetition frequency and dietary variety over the course of a week during follow-up visits while managing the KDT.

To protect children undergoing KDT from nutritional deficiencies, supplementation is widely employed, as are foods for special medical purposes fortified with vitamins and minerals [11]. However, from the perspective of the child’s lifelong health trajectory, it seems essential to make every effort to establish appropriate eating habits and preferences regarding food type and texture as early as possible, so that these may be sustained into adulthood. Dietary therapy should be conducted with a view toward its eventual discontinuation [80,81,82,83].

It appears that the variety and quality of the diet may be determined not only by carefully calculated dietary composition and balance, or by appropriate supplementation, but also by psychodietetic factors—such as food preferences, texture preferences, and food neophobia.

Ketogenic diet therapies are often perceived by parents as demanding and incompatible with children’s dietary preferences. However, children undergoing this treatment have been found to prefer higher-fat foods, in contrast to their peers, who tend to favor high-carbohydrate foods [17]. This preference does not eliminate all challenges associated with dietary implementation, as children may still prefer only specific food categories, even within permitted food groups.

Given the eating difficulties experienced by children and the impact of the KDT on the whole family, it seems appropriate to introduce targeted parental education addressing coping strategies and parenting styles, meal organization (timing, atmosphere, and dietary variety), and children’s eating behaviors. Furthermore, educational sessions should be introduced for children, focusing on techniques to reduce food neophobia and fussy eating while increasing dietary variety, particularly through hands-on experiences [84,85,86], such as cookery workshops for children and their parents. Such educational initiatives should consider dietary restrictions, the child’s health status and functioning, and parental capacity.

## 5. Strengths and Limitations

This study is the first to provide a multifaceted assessment of the dietary habits of children following KDT and to compare them with those of healthy children, which constitutes the principal novelty of this publication. The selection of both the KD and control groups was guided by the principle of homogeneity, in order to eliminate the potential influence of treatment at different centers and place of residence on dietary habits—a factor we consider to be a strength of the present study. However, this approach resulted in a limited sample size for the case group, which may have affected the results. It is worth noting that the outcomes may be partly attributable to a high degree of interindividual variability, which is more pronounced given the relatively small sample size. This, however, reflects the specific nature of research involving children with comparatively rare diseases, which are always, to some extent, shaped by the individuality of each patient.

Larger, multicenter studies are, therefore, warranted, for example, to assess the entire population of Polish patients on a KD. It seems that the relatively smaller sample size of the KD group, compared to the larger number of healthy children included in the PCA analysis, may potentially obscure patterns specific to the KD group. Consequently, future analyses involving a larger cohort of children undergoing KDT would allow for these findings to be expanded upon, confirmed, or compared with the results obtained in the present study. The current investigation may thus be regarded as a pilot study, serving as a precursor to future, larger-scale multicenter research.

The control group was designed to be representative of the general pediatric population of Lower Silesia and was selected to ensure balanced representation across age, sex, and place of residence. This approach reduced sampling randomness. However, some degree of random variation may still be a limitation of the present study.

The study employed existing questionnaires originally designed for healthy children, applied to both the KD and control groups. This constitutes a limitation of the present study to some extent. However, no dedicated questionnaires currently exist for assessing texture preferences or eating habits in children on low-carbohydrate or KDT. Moreover, the questionnaires used are characterized by their own specific granularity, which could impact result interpretation. Therefore, future studies should incorporate additional instruments to provide a broader comparative picture.

The development of such dedicated questionnaires appears highly important, particularly for children who have followed ketogenic diet therapies since early childhood or for a prolonged period, and who, for instance, have never consumed fruit juice containing pulp or are unfamiliar with the texture of other high-carbohydrate foods.

Furthermore, during the assessment of texture preferences, it was observed that children expressed a desire to consume products depicted in images that were restricted due to their high carbohydrate content. Consequently, developing questionnaires that list low-carbohydrate alternatives to conventional products appears warranted. However, in the present study, administering identical materials to both the KD and control groups enabled a direct comparison between the two cohorts. To facilitate future research involving larger cohorts of pediatric patients following KDT, it would be highly beneficial to design dietary assessment materials specifically suitable for this population.

The CEBQ was also not specifically designed for children on a KDT. However, the phrasing of the questions—utilizing expressions such as “if my child could”—made it possible to identify the preferences and habits of both groups of children. Questions phrased differently, such as those in the present tense (e.g., “my child always has something in their mouth”), would have been inappropriate for use in the case group.

## 6. Conclusions

Children with drug-resistant epilepsy following KDT do not differ significantly in most behavioral eating patterns from their peers and exhibit similarly high rates of eating difficulties, such as food neophobia and food fussiness. The present study’s findings highlight the importance of assessing tendencies toward repetitive eating, the degree of dietary diversity, and emotional eating behaviors to provide effective nutritional support during the KD treatment. Furthermore, eating habits tend to cluster into distinct patterns that predispose children to either adaptive (“easy”) or maladaptive (“difficult”) approaches to diet and food. There is a need for larger sample size studies to explore this topic in greater depth, compare our findings with those of future studies, and assess additional factors influencing the psychodietetic aspects of working with patients on the KDT. Consequently, our findings may prompt discussion on the introduction of screening assessments for food neophobia or food fussiness—utilizing tools such as the CEBQ Food Fussiness subscale or food neophobia questionnaires—to facilitate the early identification of patients at risk of developing unfavorable eating styles. Finally, they may contribute to future KDT guideline development with tailored modifications based on predominant eating behaviors.

## Figures and Tables

**Table 1:** Comparison of baseline characteristics and nutritional status between KD (*n* = 15) and control (*n* = 100) groups.

	KD Group (*n* = 15)		Control Group (*n* = 100)		KD vs. Control *p* Value	KD vs. Control Adjusted *p*-Value
sex	F	M	F	M		
	40% (n = 6)	60% (n = 9)	50% (n = 50)	50% (n = 50)	0.4699	>0.9999
place of residence >100.000/<100.000 inh.	53.33% (n = 8)	46.67% (n = 7)	50% (n = 50)	50% (n = 50)	0.8097	>0.9999
age	7 (5–9.50)		8 (6–11)		0.3811	>0.9999
centile weight	53 (31.50–78.00)		51.50 (35.50–71.25)		0.7147	>0.9999
centile weight (F/M)	49 (39.00–68.75)	67 (26.00–80.00)	48 (28.50–70.50)	59 (39.50–71.75)	0.9761	
centile height	43 (26.50–59.50)		51 (31.75–70.25)		0.4181	>0.9999
centile height (F/M)	52.5 (21.75–76.5)	43 (35.00–49.00)	51 (32.25–71.00)	47.5 (32.75–66.5)	0.0787	
centile BMI	50 (21.50–92.50)		51.50 (33.00–69.50)		0.7303	>0.9999
centile BMI (F/M)	42 (18.5–87.25)	50 (22.00–92.00)	53 (38.25–76.25)	50 (24.25–65.00)	0.3821	
underweight	0% (n = 0)		5% (n = 5)		0.0396	0.9499
underweight (F/M)	0%(n = 0)	0% (n = 0)	4% (n = 2)	6%(n = 3)	0.0637	
normal weight	66.67% (n = 10)		84% (n = 84)		0.0396	0.9499
normal weight (F/M)	66.67% (n = 4)	66.67% (n = 6)	84% (n = 42)	84%(n = 42)	0.0637	
overweight	20% (n = 3)		10% (n = 10)		0.0396	0.9499
overweight (F/M)	0% (n = 0)	33.33% (n = 3)	10%(n = 5)	10% (n = 5)	0.0637	
obesity	13.33% (n = 2)		1% (n = 1)		0.0396	0.9499
obesity (F/M)	33.33% (n = 2)	0% (n = 0)	2%(n = 1)	0% (n = 0)	0.0637	

Values presented as median (interquartile range); F, female; M, male; KD group, ketogenic diet group.

**Table 2:** Comparison of levels of food neophobia and food texture preferences between KD (*n* = 15) and control (*n* = 100) groups.

	KD Group (*n* = 15)	Control Group (*n* = 100)	*p* Value
degree of food neophobia			
lower degree of neophobia ≤ 12 pts	40.00% (n = 6)	27.00% (n = 27)	0.3605
higher degree of neophobia >12 pts	60.00% (n = 9)	73.00% (n = 73)	
>12–18 pts (>Median)	46.67% (n = 7)	54.00% (n = 54)	
>18 pts (>Q3)	13.33% (n = 2)	19.00% (n = 19)	
food texture preferences			
“soft texture likers” <25th percentile	40% (n = 6)	24% (n = 24)	0.4159
mix texture preferences 25th–75th percentile	26.67% (n = 4)	36% (n = 36)	
“hard texture likers” >75th percentile	33.33% (n = 5)	40% (n = 40)	

**Table 3:** Comparison of children’s eating behaviors (CEBQ) between KD (*n* = 15) and control (*n* = 100) groups.

		KD Group (*n* = 15)			Control Group (*n* = 100)				
	Eating Behavior	M (IRQ)	MIN	MAX	M (IRQ)	MIN	MAX	*p* Value	Adjusted *p*-Value
1.	satiety responsiveness (5–25 pts)	13 (11.5–14.5)	6	19	15(13–17)	6	21	0.0353	0.8474
2.	slowness in eating (4–20 pts)	11(8.5–13.5)	4	20	10 (8–13)	4	18	0.3325	>0.9999
3.	food fussiness (6–30 pts)	13 (10.5–20.5)	7	28	17.5 (13–22)	8	29	0.1476	>0.9999
4.	food responsiveness (5–25 pts)	11 (9–20.5)	7	25	12 (10–14.25)	5	25	0.8873	>0.9999
5.	enjoyment of food (4–20 pts)	13 (11–18)	9	20	13.5 (11–15)	6	20	0.3810	>0.9999
6.	desire to drink (3–15 pts)	7 (4- 9.5)	3	14	7 (6–9)	4	14	0.2384	>0.9999
7.	emotional undereating (4–20 pts)	4 (4–8.5)	4	16	10 (9–13)	4	20	0.0002	**0.0059**
8.	emotional overeating (4–20 pts)	4(4–7)	4	17	8 (6–10)	4	14	0.0008	**0.0196**

M (IRQ), values presented as median (interquartile range); MIN, minimum value; MAX, maximum value; pts, points; KD group, ketogenic diet group.

**Table 4:** Comparison of eating behaviors (CEBQ) levels between KD (*n* = 15) and control (*n* = 100) groups.

Eating Behavior		KD Group (*n* = 15)			Control Group (*n* = 100)		
		Low Level of Eating Style	Elevated Level of Eating Style	High Level of Eating Style	Low Level of Eating Style	Elevated Level of Eating Style	High Level of Eating Style
1.	Satiety responsiveness (5–25 pts)	40% (n = 6)	46.67% (n = 7)	13.33% (n = 2)	20% (n = 20)	58% (n = 58)	22% (n = 22)
2.	Slowness in eating (4–20 pts)	66.67% (n = 10)	6.66% (n = 1)	26.67% (n = 4)	63% (n = 63)	19% (n = 19)	18% (n = 19)
3.	Food fussiness (6–30 pts)	60% (n = 9)	26.67% (n = 4)	13.33% (n = 2)	39% (n = 39)	42% (n = 42)	19% (n = 19)
4.	Food responsiveness (5–25 pts)	40% (n = 6)	26.67% (n = 4)	33.33% (n = 5)	55% (n = 55)	30% (n = 30)	15% (n = 15)
5.	Enjoyment of food (4–20 pts)	26.67% (n = 4)	40% (n = 6)	33.33% (n = 5)	22% (n = 22)	63% (n = 63)	15% (n = 15)
6.	Desire to drink (3–15 pts)	33.33% (n = 5)	40% (n = 6)	26.67% (n = 4)	53% (n = 53)	24% (n = 24)	23% (n = 23)
7.	Emotional undereating (4–20 pts)	80% (n = 12)	13.33% (n = 2)	6.67% (n = 1)	53% (n = 53)	25% (n = 25)	22% (n = 22)
8.	Emotional overeating (4–20 pts)	93.33% (n = 14)	0% (n = 0)	6.67% (n = 1)	90% (n = 90)	0% (n = 0)	10% (n = 10)

Low level of eating style was defined as ≤50% of points for the subscale, moderate level as >50% of points and ≤M ± SD, and high level as >M ± SD (except for emotional under- and overeating). Due to the lack of significant differences between groups, the same median and SD cutoff values were used for each variable in both groups; pts, points.

## Data Availability

The data presented in this study are not publicly available due to privacy and ethical restrictions related to patient information. Data may be made available by the corresponding author upon reasonable request and with permission of the Bioethics Committee of Wroclaw Medical University.

## References

[1] Walczyk T., Wick J.Y. (2017). The Ketogenic Diet: Making a Comeback. Consult. Pharm..

[2] Simeone T.A., Simeone K.A., Rho J.M. (2017). Ketone Bodies as Anti-Seizure Agents. Neurochem. Res..

[3] Yoo B.M., Kim S.R., Lee B.-W. (2025). Ketone Body Induction: Insights into Metabolic Disease Management. Biomedicines.

[4] Martin-McGill K.J., Lambert B., Whiteley V.J., Wood S., Neal E.G., Simpson Z.R., Schoeler N.E. (2019). Understanding the core principles of a ‘modified ketogenic diet’: A UK and Ireland perspective. J. Hum. Nutr. Diet..

[5] Schoeler N.E., Whiteley V.J., Guglielmetti M., Neri L.C., Blackford R., Tan-Smith C., Aylward N., Barwick K., Caballero M.E., Gudej-Rosa S. (2025). Classical and Modified Ketogenic Diets for Children and Young People With Drug-Resistant Epilepsy: A Reflection of International Dietetic Practice and Best Practice Recommendations for Dietitians. J. Hum. Nutr. Diet..

[6] Mhanna A., Mhanna M., Beran A., Al-Chalabi M., Aladamat N., Mahfooz N. (2022). Modified Atkins diet versus ketogenic diet in children with drug-resistant epilepsy: A meta-analysis of comparative studies. Clin. Nutr. ESPEN.

[7] Poorshiri B., Barzegar M., Tahmasebi S., Shiva S., Raeisi S., Ebadi Z. (2021). The efficacy comparison of classic ketogenic diet and modified Atkins diet in children with refractory epilepsy: A clinical trial. Acta Neurol. Belg..

[8] Lambrechts D.A., de Kinderen R.J., Vles H.S., de Louw A.J., Aldenkamp A.P., Majoie M.J. (2015). The MCT-ketogenic diet as a treatment option in refractory childhood epilepsy: A prospective study with 2-year follow-up. Epilepsy Behav..

[9] Sondhi V., Agarwala A., Pandey R.M., Chakrabarty B., Jauhari P., Lodha R., Toteja G.S., Sharma S., Paul V.K., Kossoff E.H. (2020). Efficacy of Ketogenic Diet, Modified Atkins Diet, and Low Glycemic Index Therapy Diet Among Children With Drug-Resistant Epilepsy: A Randomized Clinical Trial. JAMA Pediatr..

[10] Kim S.H., Kang H.C., Lee E.J., Lee J.S., Kim H.D. (2017). Low glycemic index treatment in patients with drug-resistant epilepsy. Brain Dev..

[11] Kossoff E.H., Zupec-Kania B.A., Auvin S., Ballaban-Gil K.R., Bergqvist A.G., Blackford R., Buchhalter J.R., Caraballo R.H., Cross J.H., Dahlin M.G. (2018). Optimal clinical management of children receiving dietary therapies for epilepsy: Updated recommendations of the International Ketogenic Diet Study Group. Epilepsia Open.

[12] Winesett S.P., Bessone S.K., Kossoff E.H. (2015). The ketogenic diet in pharmacoresistant childhood epilepsy. Expert. Rev. Neurother..

[13] IJff D.M., Postulart D., Lambrechts D.A., Majoie M.H., de Kinderen R.J., Hendriksen J.G., Evers S.M., Aldenkamp A.P. (2016). Cognitive and behavioral impact of the ketogenic diet in children and adolescents with refractory epilepsy: A randomized controlled trial. Epilepsy Behav..

[14] Operto F.F., Labate A., Aiello S., Perillo C., de Simone V., Rinaldi R., Coppola G., Pastorino G.M. (2023). The Ketogenic Diet in Children with Epilepsy: A Focus on Parental Stress and Family Compliance. Nutrients.

[15] Carroll J.H., Martin-McGill K.J., Cross J.H., Hickson M., Williams E., Aldridge V., Collinson A. (2022). Core outcome set development for childhood epilepsy treated with ketogenic diet therapy: Results of a scoping review and parent interviews. Seizure.

[16] Lin A., Turner Z., Doerrer S.C., Stanfield A., Kossoff E.H. (2017). Complications During Ketogenic Diet Initiation: Prevalence, Treatment, and Influence on Seizure Outcomes. Pediatr. Neurol..

[17] Amari A., Dahlquist L., Kossoff E.H., Vining E.P., Trescher W.H., Slifer K.J. (2007). Children with seizures exhibit preferences for foods compatible with the ketogenic diet. Epilepsy Behav..

[18] Werthmann J., Jansen A., Havermans R., Nederkoorn C., Kremers S., Roefs A. (2015). Bits and pieces. Food texture influences food acceptance in young children. Appetite.

[19] Białek-Dratwa A., Kowalski O. (2023). Prevalence of Feeding Problems in Children and Associated Factors—A Cross-Sectional Study among Polish Children Aged 2–7 Years. Nutrients.

[20] Steinsbekk S., Bonneville-Roussy A., Fildes A., Llewellyn C.H., Wichstrøm L. (2017). Child and parent predictors of picky eating from preschool to school age. Int. J. Behav. Nutr. Phys. Act..

[21] Aldridge V.K., Dovey T.M., Martin C.I., Meyer C. (2016). Relative contributions of parent-perceived child characteristics to variation in child feeding behavior. Infant Ment. Health J..

[22] Kutbi H.A., Alhatmi A.A., Alsulami M.H., Alghamdi S.S., Albagar S.M., Mumena W.A., Mosli R.H. (2019). Food neophobia and pickiness among children and associations with socioenvironmental and cognitive factors. Appetite.

[23] Gibson E.L., Cooke L. (2017). Understanding Food Fussiness and Its Implications for Food Choice, Health, Weight and Interventions in Young Children: The Impact of Professor Jane Wardle. Curr. Obes. Rep..

[24] Lafraire J., Rioux C., Giboreau A., Picard D. (2016). Food rejections in children: Cognitive and social/environmental factors involved in food neophobia and picky/fussy eating behavior. Appetite.

[25] Dovey T.M., Staples P.A., Gibson E.L., Halford J.C. (2008). Food neophobia and ‘picky/fussy’ eating in children: A review. Appetite.

[26] Pliner P. (1994). Development of Measures of Food Neophobia in Children. Appetite.

[27] Harris G., Reilly S. (2018). Food neophobia: Behavioral and biological influences: Neophobia at 20 months: A visual categorization problem?. Food Neophobia.

[28] Wolstenholme H., Kelly C., Hennessy M., Heary C. (2020). Childhood fussy/picky eating behaviours: A systematic review and synthesis of qualitative studies. Int. J. Behav. Nutr. Phys. Act..

[29] Taylor C.M., Wernimont S.M., Northstone K., Emmett P.M. (2015). Picky/fussy eating in children: Review of definitions, assessment, prevalence and dietary intakes. Appetite.

[30] Trofholz A.C., Schulte A.K., Berge J.M. (2017). How parents describe picky eating and its impact on family meals: A qualitative analysis. Appetite.

[31] Finistrella V., Gianni N., Fintini D., Menghini D., Amendola S., Donini L.M., Manco M. (2024). Neophobia, sensory experience and child’s schemata contribute to food choices. Eat. Weight Disord..

[32] Laureati M., Sandvik P., Almli V.L., Sandell M., Zeinstra G.G., Methven L., Wallner M., Jilani H., Alfaro B., Proserpio C. (2020). Individual differences in texture preferences among European children: Development and validation of the Child Food Texture Preference Questionnaire (CFTPQ). Food Qual. Prefer..

[33] Brown C.L., Vander Schaaf E.B., Cohen G.M., Irby M.B., Skelton J.A. (2016). Association of Picky Eating and Food Neophobia with Weight: A Systematic Review. Child. Obes..

[34] Falciglia G.A., Couch S.C., Gribble L.S., Pabst S.M., Frank R. (2000). Food Neophobia in Childhood Affects Dietary Variety. J. Am. Diet. Assoc..

[35] Galloway A.T., Fiorito L., Lee Y., Birch L.L. (2005). Parental pressure, dietary patterns, and weight status among girls who are “picky eaters”. J. Am. Diet. Assoc..

[36] Guzek D., Głąbska D., Lange E., Jeżewska-Żychowicz M. (2017). A Polish Study on the Influence of Food Neophobia in Children (10–12 Years Old) on the Intake of Vegetables and Fruits. Nutrients.

[37] Hawkes C.P., Roy S.M., Dekelbab B., Frazier B., Grover M., Haidet J., Listman J., Madsen S., Roan M., Rodd C. (2021). Hypercalcemia in Children Using the Ketogenic Diet: A Multicenter Study. J. Clin. Endocrinol. Metab..

[38] Corsello A., Trovato C.M., Di Profio E., Cardile S., Campoy C., Zuccotti G., Verduci E., Diamanti A. (2023). Ketogenic diet in children and adolescents: The effects on growth and nutritional status. Pharmacol. Res..

[39] Mendonça C.N., Henriques-Souza A.M., Viana L.A., Souza P.A., Alves Neto L.B., Mello M.J. (2024). Ketogenic diet in pharmacoresistant epilepsies: A clinical nutritional assessment. Arq. Neuropsiquiatr..

[40] Mustafa M.S., Shafique M.A., Aheed B., Ashraf F., Ali S.M., Iqbal M.F., Haseeb A. (2024). The impact of ketogenic diet on drug-resistant epilepsy in children: A comprehensive review and meta-analysis. Ir. J. Med. Sci..

[41] Armeno M., Verini A., Del Pino M., Araujo M.B., Mestre G., Reyes G., Caraballo R.H. (2019). A Prospective Study on Changes in Nutritional Status and Growth Following Two Years of Ketogenic Diet (KD) Therapy in Children with Refractory Epilepsy. Nutrients.

[42] Santiworakul C., Chomtho K., Chomtho S. (2021). Growth and nutritional status of pediatric patients treated with the ketogenic diet. Asia Pac. J. Clin. Nutr..

[43] Kułaga Z., Różdżyńska-Świątkowska A., Grajda A., Gurzkowska B., Wojtyło M., Góźdż M., Świąder-Leśniak A., Litwin M. (2015). Percentile charts for growth and nutritional status assessment in Polish children and adolescents from birth to 18 year of age. Stand. Med. Pediatr..

[44] An M., Zhou Q., Younger K.M., Liu X., Kearney J.M. (2020). Are Maternal Feeding Practices and Mealtime Emotions Associated with Toddlers’ Food Neophobia? A Follow-Up to the DIT-Coombe Hospital Birth Cohort in Ireland. Int. J. Environ. Res. Public Health.

[45] Cooke L., Carnell S., Wardle J. (2006). Food neophobia and mealtime food consumption in 4–5 year old children. Int. J. Behav. Nutr. Phys. Act..

[46] Cooke L.J., Wardle J., Gibson E.L., Sapochnik M., Sheiham A., Lawson M. (2004). Demographic, familial and trait predictors of fruit and vegetable consumption by pre-school children. Public Health Nutr..

[47] Damsbo-Svendsen M., Frøst M.B., Olsen A. (2017). A review of instruments developed to measure food neophobia. Appetite.

[48] Wardle J., Guthrie C.A., Sanderson S., Rapoport L. (2001). Development of the Children’s Eating Behaviour Questionnaire. J. Child Psychol. Psychiatry.

[49] Cappellotto M., Olsen A. (2021). Food Texture Acceptance, Sensory Sensitivity, and Food Neophobia in Children and Their Parents. Foods.

[50] Kozioł-Kozakowska A., Piórecka B., Schlegel-Zawadzka M. (2018). Prevalence of food neophobia in pre-school children from southern Poland and its association with eating habits, dietary intake and anthropometric parameters: A cross-sectional study. Public Health Nutr..

[51] Sleddens E.F., Kremers S.P., Thijs C. (2008). The children’s eating behaviour questionnaire: Factorial validity and association with Body Mass Index in Dutch children aged 6–7. Int. J. Behav. Nutr. Phys. Act..

[52] Malczyk Ż., Kuczka O., Pasztak-Opiłka A., Zachurzok A. (2022). Validation of the Children’s Eating Behaviour Questionnaire in Poland. Nutrients.

[53] Han R., Xu Y., Sun J., Guo Q., Du K., Dong Y., Li X., Zhang L., Chen H., Li L. (2026). Real-world outcomes of ketogenic diet therapy in children with drug-resistant epilepsy in a prospective cohort study. Sci. Rep..

[54] Chang J., Schimpf S., Phitsanuwong C. (2025). Impact of modified Atkins diet on growth in infants and children with epilepsy. Epileptic Disord..

[55] Lu S., Champion H., Mills N., Simpson Z., Whiteley V.J., Schoeler N.E. (2023). Impact of ketogenic diet therapy on growth in children with epilepsy. Epilepsy Res..

[56] Białek-Dratwa A., Kowalski O. (2024). Feeding neophobia and current feeding problems—A cross-sectional study among Polish children aged 2–7 years. Pediatr. Pol..

[57] Zou J., Liu Y., Yang Q., Liu H., Luo J., Ouyang Y., Wang J., Lin Q. (2019). Cross-cultural adaption and validation of the Chinese version of the Child Food Neophobia Scale. BMJ Open.

[58] Piochi M., Fino M.A., Torri L. (2025). The Impact of Children’s Food Neophobia on Meal Perception, Emotional Responses, and Food Waste in Italian Primary School Canteens. Foods.

[59] Białek-Dratwa A., Szczepańska E., Szymańska D., Grajek M., Krupa-Kotara K., Kowalski O. (2022). Neophobia—A Natural Developmental Stage or Feeding Difficulties for Children?. Nutrients.

[60] Johnson S.L., Davies P.L., Boles R.E., Gavin W.J., Bellows L.L. (2015). Young Children’s Food Neophobia Characteristics and Sensory Behaviors Are Related to Their Food Intake. J. Nutr..

[61] Krupa-Kotara K., Nowak B., Markowski J., Rozmiarek M., Grajek M. (2024). Food Neophobia in Children Aged 1–6 Years—Between Disorder and Autonomy: Assessment of Food Preferences and Eating Patterns. Nutrients.

[62] Faith M.S., Heo M., Keller K.L., Pietrobelli A. (2013). Child food neophobia is heritable, associated with less compliant eating, and moderates familial resemblance for BMI. Obesity.

[63] Dipasquale V., Cucinotta U., Alibrandi A., Laganà F., Ramistella V., Romano C. (2023). Early Tube Feeding Improves Nutritional Outcomes in Children with Neurological Disabilities: A Retrospective Cohort Study. Nutrients.

[64] Chow C.Y., Skouw S., Bech A.C., Olsen A., Bredie W.L. (2024). A review on children’s oral texture perception and preferences in foods. Crit. Rev. Food Sci. Nutr..

[65] van der Louw E., van den Hurk D., Neal E., Leiendecker B., Fitzsimmon G., Dority L., Thompson L., Marchió M., Dudzińska M., Dressler A. (2016). Ketogenic diet guidelines for infants with refractory epilepsy. Eur. J. Paediatr. Neurol..

[66] Malczyk Ż., Pasztak-Opiłka A., Zachurzok A. (2024). Different Eating Habits Are Observed in Overweight and Obese Children Than in Normal-Weight Peers. Children.

[67] Masento N.A., Dulay K.M., Harvey K., Bulgarelli D., Caputi M., Cerrato G., Molina P., Wojtkowska K., Pruszczak D., Barlińska J. (2022). Parent, child, and environmental predictors of vegetable consumption in Italian, Polish, and British preschoolers. Front. Nutr..

[68] Lambrechts D.A., Bovens M.J., de la Parra N.M., Hendriksen J.G., Aldenkamp A.P., Majoie M.J. (2013). Ketogenic diet effects on cognition, mood, and psychosocial adjustment in children. Acta Neurol. Scand..

[69] Varesio C., Zanaboni M.P., Pasca L., Provenzi L., Ferraris C., Tagliabue A., Pezzotti E., Carpani A., Veggiotti P., De Giorgis V. (2024). Novel insight into GLUT1 deficiency syndrome: Screening for emotional and behavioral problems in youths following ketogenic diet. Minerva Pediatr..

[70] Stone R.A., Blissett J., Haycraft E., Farrow C. (2022). Predicting preschool children’s emotional eating: The role of parents’ emotional eating, feeding practices and child temperament. Matern. Child Nutr..

[71] Mentzelou M., Papadopoulou S.K., Psara E., Alexatou O., Koimtsidis T., Giaginis C. (2025). Exploring the Impact of Emotional Eating in Children: A Narrative Review. Pediatr. Rep..

[72] Zborowski M., Skotnicka M. (2025). The Role of Hydration in Children and Adolescents—A Theoretical Framework for Reviewing Recommendations, Models, and Empirical Studies. Nutrients.

[73] Pjetraj D., Pjetraj A., Sayed D., Severini M., Falcioni L., Svarca L.E., Gatti S., Lionetti M.E. (2025). Decoding Picky Eating in Children: A Temporary Phase or a Hidden Health Concern?. Nutrients.

[74] Fildes A., Mallan K.M., Cooke L., van Jaarsveld C.H., Llewellyn C.H., Fisher A., Daniels L. (2015). The relationship between appetite and food preferences in British and Australian children. Int. J. Behav. Nutr. Phys. Act..

[75] Di Nucci A., Pilloni S., Scognamiglio U., Rossi L. (2023). Adherence to Mediterranean Diet and Food Neophobia Occurrence in Children: A Study Carried out in Italy. Nutrients.

[76] Del Campo C., Bouzas C., Tur J.A. (2024). Risk Factors and Consequences of Food Neophobia and Pickiness in Children and Adolescents: A Systematic Review. Foods.

[77] Björelius H., Tsilingaridis G., Johansson F., Trang J., Grigoriadis A., Thorman R., Terband H. (2026). Chewing efficiency in children with motor speech disorders. Eur. Arch. Paediatr. Dent..

[78] Le Révérend B.J., Edelson L.R., Loret C. (2014). Anatomical, functional, physiological and behavioural aspects of the development of mastication in early childhood. Br. J. Nutr..

[79] Kollia B., Tsiamtsiouris J., Korik P. (2019). Oral motor treatment: Effects of therapeutic feeding on articulatory skills. J. Prev. Interv. Community.

[80] Issanchou S., Habeat Consortium (2017). Determining Factors and Critical Periods in the Formation of Eating Habits: Results from the Habeat Project. Ann. Nutr. Metab..

[81] Craigie A.M., Lake A.A., Kelly S.A., Adamson A.J., Mathers J.C. (2011). Tracking of obesity-related behaviours from childhood to adulthood: A systematic review. Maturitas.

[82] Tarro S., Vahtera J., Pentti J., Niinikoski H., Raitakari O., Rönnemaa T., Viikari J., Pahkala K., Lagström H. (2025). Diet Quality Trajectories from Infancy to Young Adulthood: The Special Turku Coronary Risk Factor Intervention Project (STRIP) Study. J. Nutr..

[83] Movassagh E.Z., Baxter-Jones A.D.G., Kontulainen S., Whiting S.J., Vatanparast H. (2017). Tracking Dietary Patterns over 20 Years from Childhood through Adolescence into Young Adulthood: The Saskatchewan Pediatric Bone Mineral Accrual Study. Nutrients.

[84] Coulthard H., Sealy A. (2017). Play with your food! Sensory play is associated with tasting of fruits and vegetables in preschool children. Appetite.

[85] Ishikawa M., Eto K., Miyoshi M., Yokoyama T., Haraikawa M., Yoshiike N. (2019). Parent-child cooking meal together may relate to parental concerns about the diets of their toddlers and preschoolers: A cross-sectional analysis in Japan. Nutr. J..

[86] DeCosta P., Møller P., Frøst M.B., Olsen A. (2017). Changing children’s eating behaviour—A review of experimental research. Appetite.

